# Modeling amyloids in bacteria

**DOI:** 10.1186/1475-2859-11-166

**Published:** 2012-12-28

**Authors:** Anna Villar-Piqué, Salvador Ventura

**Affiliations:** 1 Departament de Bioquímica i Biologia Molecular, Facultat de Biociències, Universitat Autònoma de Barcelona, Bellaterra E-08193, Spain; 2 Institut de Biotecnologia i de Biomedicina, Universitat Autònoma de Barcelona, Bellaterra E-08193, Spain

## Abstract

An increasing number of proteins are being shown to assemble into amyloid structures, self-seeding fibrillar aggregates that may lead to pathological states or play essential biological functions in organisms. Bacterial cell factories have raised as privileged model systems to understand the mechanisms behind amyloid assembly and the cellular fitness cost associated to the formation of these aggregates. In the near future, these bacterial systems will allow implementing high-throughput screening approaches to identify effective modulators of amyloid aggregation.

## 

The aggregation of proteins into amyloid structures is the triggering event on the onset of a growing number of human disorders, from neurodegenerative diseases as Alzheimer, Parkinson, Huntington or transmissible spongiform encephalopathies to non-neurodegenerative systemic and localized amyloidosis, as senile systemic amyloidosis or type II diabetes [1]. In addition, it is now clear that different organisms, from virus to humans, exploit the special architecture of amyloids for functional purposes, such as cellular invasion or hormone storage [2]. Therefore, protein aggregation has emerged from a neglected area of protein science to a central issue in biology and biomedicine.

Many biochemical pathways, from DNA replication to protein degradation, have been modeled first in bacteria. However, despite it has been long recognized that heterologous protein expression in bacterial cell factories results often in the formation of insoluble deposits composed essentially by the target protein [3,4], only recently some groups have dared to exploit this well-characterized phenomena to model amyloid formation [5,6]. This delay resulted mainly from the fact that these aggregates, known as inclusion bodies (IBs), were traditionally considered unstructured protein particles only useful to obtain denatured protein for refolding purposes. This old framework has changed into a new scenario where intracellular aggregation in bacteria is providing important clues on the molecular determinants of amyloid formation and its remediation [7,8].

A first inflection in the field came along with early reports describing a selective molecular structure inside IBs. It has been found a substantial similitude between the properties of these bacterial aggregates and the pathological fibrils linked to amyloidosis. On one side, IBs generally bind thioflavin T and Congo Red, the typical amyloid dyes, and display seeding ability in the fibrillar assembly of homologous monomers [9,10]. On the other side, low resolution techniques, such as infra-red spectroscopy, circular dichroism or X-ray difracction, denote the presence of signals corresponding to tightly packed intermolecular β-sheets, similar to those in amyloid fibrils [9-11]. Importantly, these findings come from independent studies using completely different protein models, not related in sequence or structure, thus suggesting that the amyloid signature might be a generic feature of bacterial aggregates. Furthermore, high resolution approaches, such as hydrogen/deuterium exchange by NMR or solid-state NMR have been used to study different bacterial IBs, defining their molecular structure at the residue level. These analysis prove that, at least for amyloidogenic proteins, bacterial IBs and fibrils share the same amyloid core. However, they also show that part of the polypeptide sequence or, alternatively, a fraction of the molecules remain disordered and/or in native-like conformations inside these aggregates [11,12]. Contrary to the previous assumption that IBs were totally inactive, the presence of native-like structure endorse IBs with a certain degree of biological activity [13-17]. This observation has opened the door to the use of bacteria as small factories to produce promising functional materials and catalysts, boosting the investigation of the structural and functional properties of IBs [18-23].

It is now clear that they are the oligomeric assemblies populating the fibrillation pathway and not the mature fibrils that exert the main cytotoxic effect in conformational disorders [2]. The structures of these oligomeric intermediates states have been the subject of debate for many years. The similarity between IBs and amyloids has arisen a critical question: do IBs exert a cytotoxic effect analogous to that recurrently observed for fibrils? The answer is: Yes, the addition of purified bacterial IBs to neuronal cultured cells produces a loss in cell viability equal to that promoted by the same concentration of amyloid material [24,25]. Bacterial IBs contain small heat shock proteins (sHSPs), which are highly homologous to those found in the aggregates of the brains of patients suffering different neuronal pathologies. It has been proposed that in the brain these sHSPs might break down amyloid fibril structure, resulting in the accumulation of toxic oligomeric species. The observation that the neurotoxicity of IBs correlates with the amount of oligomeric assemblies and chaperones in these aggregates and the possibility to identify at the residue level the determinants of this effect [25] are expected to provide new molecular insights on the structures of the deleterious species in amyloid assemblies. Thus, protein overexpression in bacterial cell factories, by mimicking the conditions in the cell under stress, will likely allow to address aspects of amyloid biology that are otherwise technically impossible to study in more complex contexts.

Prion proteins are a particularly interesting and dangerous type of amyloids, since their aggregated states have self-perpetuating ability and thus become infectious. Het-s, from the fungus *Podospora anserina*, was the first prion protein whose bacterial IBs were shown to display amyloid-like properties [12,26]. The differential trait of these aggregates emerged when they were transfected into prion-free fungal strains, as they promoted prionic conversion at levels comparable to those induced by homologous amyloid fibrils [12]. This result has been later corroborated in the case of the yeast prion Sup35. The IBs of this protein have been used to induce the prion phenotype in prion-free yeast strains, with the novel evidence that the infectivity rate can be easily modulated by tuning the environmental conditions during the formation of IBs [27]. When instead of being expressed intracellularly, this protein is directed to the secretory pathway, the aggregates are formed in the cell surface of bacteria, but they are also able to template the conformational prionic change [28]. These recent observations provide perhaps the best confirmation that the IBs molecular structure highly resembles to the fine architecture of amyloid fibrils, in such a way that even the infectious properties of amyloids, which depend on very specific conformational properties, are conserved in the two type of aggregates. This evidence bears important implications for the use of bacteria to model amyloids, since prion-like behaviour is currently receiving preferential attention in the field, due to the growing realisation that protein-based infection may be behind frequently occurring neurodegenerative disorders such Alzheimer’s and Parkinson’s diseases [29].

The increasing medical and economic impact of aggregation-linked diseases in our society has fueled the development of methods to identify chemical compounds that can interfere with amyloidogenic pathways, having thus therapeutic potential to treat or prevent these disorders. Generally, these assays, used by many biopharma companies, are cumbersome, lack reproducibility, use expensive synthetic peptides and are performed in physiologically non-relevant contexts. Several labs are focusing their efforts towards bacterial systems to overcome these limitations. In this context, fluorescent tag reporters of aggregation have been employed in bacteria to measure in a straightforward manner the amyloid assembly rate, as the final fluorescence of the aggregate is the result of a kinetic competition between folding and aggregation [15,30,31]. Any compound that enhances or inhibits one of these two competing reactions can be easily detected by spectrofluorometry. This property has been exploited recently both in living bacteria and *in vitro,* using purified IBs, to implement high-throughput, 96-well-plate based, assays able to identify and characterize novel amyloid modulators in large compound libraries [32,33]. As stated above, the amyloid nature of bacterial aggregates can be assessed using dyes such as Thioflavin-S (Th-S), whose spectroscopic properties change upon binding to amyloid structures. This characteristic, together with the ability of the dye to enter intact cells can be used to detect *in vivo* the formation of amyloid structures inside bacteria. The application of flow cytometry to detect (Th-S) fluorescence has been shown to be a fast, robust, quantitative, non-invasive method to screen for the presence of *in vivo* intracellular amyloid-like aggregates in bacteria as well as for monitoring the effect of amyloid inhibitors in intact cells, skipping the need for a genetically encoded reporter [34]. Although still in an early stage, it is clear that, apart from its academic interest, modeling amyloid formation in bacteria might render important economic revenues. The above examples illustrate how bacterial cell factories can be easily adapted to develop screening tools for amyloid aggregation inhibitors that will outperform the conventional screening procedures used by the industry.
